# From Phosphorous to Arsenic: Changing the Classic Paradigm for the Structure of Biomolecules

**DOI:** 10.3390/biom2020282

**Published:** 2012-05-30

**Authors:** Ryan Knodle, Pratima Agarwal, Mark Brown

**Affiliations:** 1 Department of Microbiology, Colorado State University, Fort Collins, CO 80523-1052, USA; Email: knodle@rams.colostate.edu; 2 Department of Biology, Boston University, Boston, MA 02215, USA; Email: pratima2@bu.edu; 3 Department of Clinical Sciences, Colorado State University, Fort Collins, CO 80523-1052, USA

**Keywords:** arsenic, GFAJ-1, biomolecules, scientific debate, elemental substitution

## Abstract

Biomolecules are composed primarily of the elements carbon, nitrogen, hydrogen, oxygen, sulfur, and phosphorus. The structured assembly of these elements forms the basis for proteins, nucleic acids and lipids. However, the recent discovery of a new bacterium, strain GFAJ-1 of the *Halomonadaceae*, has shaken the classic paradigms for the architecture of life. Mounting evidence supports the claim that these bacteria substitute arsenic for phosphorus in macromolecules. Herein, we provide a brief commentary and fuel the debate related to what may be a most unusual organism.

## 1. Introduction

There exists a wonderful dichotomy in science. On one hand, we have universal laws and principles that are constant: the force of gravity; Newton’s laws of motion; the principles of thermodynamics. On the other hand, scientists discover new things on a daily basis, from the “discovery of the accelerating expansion of the universe through observations of distant supernova” [1] which is incomprehensibly large compared to humankind, to the discovery of quasicrystals in chemistry, which exist on the atomic level. Every once in a while, these new discoveries uproot notions of our world which have existed for hundreds of years, effectively redefining what we understand about the natural world. As such, science has traditionally held that life depends on six essential bioelements: carbon, hydrogen, oxygen, nitrogen, sulfur, and phosphorus. These bioelements, with the help of trace elements - namely metals and metalloids - compose all lipids, proteins, and nucleic acids and thus, are the building blocks of life. The complex interactions between molecules, until recently, were thought to be intricate to the point that there could not be any deviation from the classic norm. Dr. Felisa Wolfe-Simon and her recent discovery of a strain of bacteria which may substitute arsenic for phosphorous in biomolecules challenge this norm.

## 2. Discussion

Since the physical constructs of life are based upon essential bioelements, scientists discerned that these six specific elements must be used without exception. Although it is broadly accepted that six aforementioned elements form the basis of all biomolecules, examples of substitutions with trace elements are not absent in the literature. For instance, copper can take the place of iron in certain mollusks and arthropods to facilitate oxygen transport. Cadmium can substitute for zinc in enzyme families [2]. In both of these examples, the substituting element shares a number of commonalities with the parent element, namely orbital configuration, electronegativity, and quantity of valence electrons. These similarities are largely due to the elements belonging to the same periodic group. In consideration of this unique relationship between trace elements, some have hypothesized that the major bioelements may be interchangeable as well. Dr. Wolfe-Simon’s discovery in California may provide evidence to support these hypotheses.

While working in Mono Lake, California, a hypersaline and alkaline lake, Dr. Wolfe-Simon stumbled upon a new bacterium; one, she argues, that can use arsenic in place of phosphorus in all of its metabolic functions. Arsenic is a chemical analog of phosphorus, just as copper is to iron, mentioned above. It sits just below phosphorus on the periodic table, making it a non-metal, and implicating similar atomic radii, the same number of valence electrons, and nearly identical electronegativity and orbital configurations. The similarity between phosphorus and arsenic translates to other molecules they form, importantly phosphate (PO_4_^3−^) and arsenate (AsO_4_^3−^). It is the similarity between these two derivatives that makes arsenic dangerous; thus its implication in many infamous homicides and questionable deaths over the last 2000 years. 

Evidence of the similarity between the structure, synthesis, and hydrolysis of both arsenate and phosphate-containing compounds is described by Moore in a study of ADP-arsenate. She cites that the equilibrium constants for the synthesis of both arsenate and phosphate esters is almost identical, implicating that such similarities in enzymatic activity show an intimate relationship between the two derivatives. The study also posits that the rate constant for both the synthesis and hydrolysis of glucose-6-arsenate is 10^5^-fold larger than the rate constant for the same reactions in phosphate, indicating that cellular processes actually favor the use of arsenate-containing compounds to that of the phosphate counterpart [3].

Arsenic and phosphorus derivatives are so similar to each other, that arsenate can be incorporated into metabolic pathways usually requiring phosphate. The consequences of this substitution are not felt until the later stages of these pathways, as arsenate is quite unstable, compared to phosphate. A notable example of the problems with arsenate substitution is seen in glycolysis, where the interchanging of arsenate forms products that easily hydrolyze to form the next intermediate which consequently inhibits the production of ATP molecules typically formed in this reaction. Due to this, arsenate is an “uncoupler of glycolysis”, which has major energy consequences [4]. Arsenate can also disrupt the conversion of pyruvate to acetyl CoA, preventing the initiation of the Krebs cycle, leading to additional loss of precious energy molecules [5]. This is the primary cause of arsenic’s toxicity.

Resistance to arsenic, especially in bacterial isolates, is not unheard of. A 2010 study by Huang *et al*. indicated that twelve isolates from various families including *Pseudomonas*, *Naxibacter*, *Acinetobacter*, *Mesorhizobium*, *Enterobacter*, *Methylobacterium*, *Bacillus*, and *Caryophanon* displayed arsenic resistance in contaminated soils around *Pteris vittata* habitats. *P. vittata*, a Chinese brake fern, is considered an arsenic hyperaccumulator which tolerates levels of arsenic significantly higher than most plants. Its tolerance of markedly increased levels of arsenic and related compounds is a result of the symbiotic relationship between the plant roots and the microbes that colonize the rhizosphere surrounding the plant [6].

The isolates mentioned above are not only arsenic resistant, but three of the isolates - one each of the *Naxibacter*, *Mesorhizobium*, *andPseudomonas* species - were shown to reduce arsenate to arsenite due to the presence of a reduction-detoxification mechanism coded by the *arr* operon. Not only does arsenic resistance occur in microbes adapted to hyperarsenic environment, but metabolic processing of arsenic has been shown in select isolates [6].

If an organism had such appropriate mechanisms as a reduction-detoxification mechanism as seen in the Huang study [6] to cope with the instability of arsenic and its derivative compounds, especially considering the known examples of trace element substitution, arsenic could be used in the place of phosphorus. This is exactly what Dr. Wolfe-Simon and her research team set out to test. Operating under the impression that organisms found in Mono Lake, a lake with atypical chemical conditions-namely high levels of arsenic, may be unique in terms of cellular processes and adaptations, the research team isolated GFAJ-1, a bacterium from the family *Halomonadaceae* [1]. In the laboratory, Wolfe-Simon and her team worked to mimic the natural environment of the bacterium by introducing lake sediments into an artificial medium at pH 9.8, supplementing with glucose, trace minerals, and vitamins, and adding specified concentrations of arsenate ranging from 100 μM–5 mM. The team did not add phosphate to the medium, and went through multiple dilution transfers to reduce any potential residual phosphorus. The medium contained slight amounts of phosphate (3 μM), the result of trace impurities in the added nutrients. The negative control in the experiment would later render this remaining phosphate negligible in terms of supporting bacterial growth [2].

Wolfe-Simon began growing the bacterium on agar plates and transferred a colony to the artificial medium described above. Progressively increasing the concentration of arsenate to identify the optimal level for growth in GFAJ-1, she found the optimal concentration is 40 mM AsO_4_^3−^ with no added phosphate. Interestingly, the bacterium grew and propagated at increased rates when phosphate was added, but no growth was observed when the colony was added to a plate with no added phosphate or arsenate. This data is crucial, because it suggests that GFAJ-1 exhibits arsenic-dependent growth but is not an obligate arsenophile [2].

In order to discern whether or not the bacterium was taking AsO_4_^3−^ from the medium, the research team measured the amount of intracellular arsenic through inductively coupled plasma mass spectrometry, a type of spectrometry capable of detecting concentrations of metals below one part per trillion (10^−12^) [7]. The bacterium displayed 10× the amount of arsenic as phosphorus in the +As/−P -grown cells. The detected phosphorus is likely the result of scavenged phosphorus from trace phosphate impurities in the reagents. The levels of residual phosphorus were far below the minimum amount needed to support growth, indicating once again, that this presence of phosphorus was not sufficient to support bacterial growth. These results were confirmed with x-ray analyses and high-resolution secondary ion mass spectrometry [2].

After confirming the presence of arsenic inside the bacterium, Dr. Wolfe-Simon investigated the intracellular location of the arsenic. Using radiolabeled arsenate, her team observed arsenic in lipids, proteins, and nucleic acids, indicating much more than just an intracellular accumulation of arsenate. Further analyses that considered the bond lengths between arsenic and other atoms as a comparison to normal bond lengths between phosphorus and those atoms confirmed integration into various cellular biomolecules [2].

So why is this significant? What could the discovery of a bacterium named with the acronym for “Give Felisa A Job” mean for science? The answer is simple: everything. This discovery, if corroborated and further studied, would be absolutely groundbreaking to all of science. This seemingly small substitution of arsenic for phosphorus would shake the pillars that all of life science sits upon. Not only would this discovery open the door up to the search for thousands if not millions of other species that can make said substitutions, but it would redefine the principles of biology that have been taught for decades. Since the discovery of DNA and the double helix by Watson and Crick with the help of Rosalind Franklin in 1953, scientists have recognized the code of life as being stable, but extremely specific. The nucleotide bases, the non-covalent interactions that allow for base-specific bonding, the 10.5 bases per helical turn, the rigid and stable backbone, and the sugar pucker all lend to the notion of maintaining an excruciatingly specific composition. The substitution of arsenic for phosphorus and its subsequent change from phosphate to arsenate changes all of that. The effects span from nucleic acids to energy molecules to the exoskeletons of chitinous arthropods and bones of animals [8].

Due to the extensive implications of the information published in Dr. Wolfe-Simon’s paper, a multitude of critics have arisen to object to the findings. Many have disagreed with the claims of arsenic substitution made in the publication, citing errors in testing ranging from improperly performing tests to gathering insufficient data from the lack of testing. Others have pointed out problems with the controls set up by the team, indicating that presence of any phosphate in the media disqualified the results. Still, others have debated whether the intracellular arsenic has been incorporated into the biomolecules or simply present in vacuoles within the bacterium [9]. At any rate, members outside of the scientific community may view the criticisms and other events that have transpired as superfluous, vindictive, and outright scathing.

Dr. Wolfe-Smith responded to many of these criticisms in interviews with various scientific and media groups. She explained that her team published the data they had at such a quick rate because her laboratory did not have all of the necessary tools to perform many of the more intensive tests that critics have called upon. By publishing the information as quickly as possible, she hoped that other labs, now with their curiosity piqued, would come forward seeking to collaborate further on this project. She has also argued that the tests performed in the published manuscript are indeed sound, and calls upon others to replicate her results [10].

It is no surprise that this discovery has come under what some may consider a brutal attack over the last year; the proposed repercussions almost beg of it. This experiment has the potential to change a dogma of biology: that the original six bioelements are essential to life. The current discussion contours that of the one surrounding the realization that prions do not follow the same biochemical patterns that are requisite of all other life. Whereas all organisms, from humans to obligate intracellular parasites - like viruses - follow the DNA to RNA to protein pathway, prions are hypothesized to contain no nucleic acids, and replicate solely with proteins. This breaks what is heralded as the central dogma of biology, and was subjected to heavy attacks from the scientific community at large when Tikvah Alper and John Griffith brought prions to the scientific community in the early 1960’s.

Dr. Wolfe-Simon’s discovery faces the same scrutiny today; just as Alper and Griffith in 1963, Darwin in 1859, and Copernicus in 1543. This discussion and debate is the culmination of science. Science is defined as the observation and testing of natural phenomena, but requires so much more than that. Without attacks heralded by intellectuals from a multitude of disciplines and the ensuing pursuit of evidence and answers, breakthroughs in science do not occur. To accept postulations and hypotheses of this magnitude without requesting - if not demanding - further research and explanation is irresponsible to the field of science. Scientific discovery hinges on discussion among peers, it always has and it always will. Those things characterized as “great scientific achievements” have withstood years and sometimes decades of criticisms. They have been accompanied by banishment, disgrace, and legal repercussions for the respective proposers. They have been defended tooth and nail from factions of the religious, scientific, and philosophical communities. Unfortunately, some have died as a result of these discussions, and the majority of intellectuals who had hands in bringing about these scientific breakthroughs died long before the debates were settled and their hypotheses deemed accurate.

## 3. Conclusions

What does all this mean for the reader? Dr. Wolfe-Simon is being subjected to the same procedure as all those who have come before her. Her hypothesis is certainly one that raises numerous questions and demands more evidence, but the implications of it have the potential to shake the foundation of biology as we have known it for centuries. We encourage continued debate, discussion, and research. We acknowledge that we may not have answers for another two, or five, or ten years, but we trust that science will operate successfully as it has for thousands of years: through healthy debate and discussion.
